# A Controlling Authority for Voluntary Hospitals
**The Functions of the Voluntary Hospitals in Relation to the Proposed Public Assistance Authority.* By J. C. Buchanan. London : Bradbury and Agnew. Price 1s. net.


**Published:** 1911-03-04

**Authors:** 


### Special Institutional Article.

### A CONTROLLING AUTHORITY FOR VOLUNTARY HOSPITALS.*

### MR. BUCHANAN'S SUGGESTIONS.

The peculiar difficulties in the administration of voluntary hospitals have been the subject of much discussion of late; the literature on the questions involved is increasing in volume and force. In a recently-issued pamphlet, Mr. J. Courtney Buchanan has embodied the remarks he made last year before the Incorporated Association of Hospital Officers on certain aspects of the problems connected with the functions of the voluntary hospitals in relation to the proposed Public Assistance Authority. It is impossible wholly to disconnect the problems of the existing voluntary charities from the concurrent movement and unrest so apparent now in the world of Poor Law Administration, but it is hoped by many, if not by most, that the time is still far distant when the voluntary system will be involved or submerged in some all-embracing scheme of Public Assistance or municipal control. The continued performance of their proper function by the voluntary charities is intimately bound up with the question of subscriptions and their tendency to diminish. The causes assigned by Mr. Buchanan to account for this will be generally admitted, so here we need only quote the conclusion which is the outcome of his remarks: "If hospitals are still to do the same work that they have hitherto done, they must sooner or later have some form of State aid or municipal subvention."

#### State-aided Hospitals Not Necessarily Inevitable.

He is, however, careful to explain that there are means of preventing this result, and he believes that many of the financial difficulties of voluntary hospitals can be relieved by a change in the method of their working as regards both in-patients and out-patients, and that the changes proposed in his paper will obviate any necessity for State aid or municipal subvention, leaving the hospitals free to do more research work. Legislation will be required, but the medical staffs would be far less disinclined to have their cases sorted in provident dispensaries outside hospitals if only the dispensaries were under consultants and if the clinical material in Poor Law and Public Assistance institutions were available for teaching purposes.

#### Wanted—A Central Hospital Council.

A considerable portion of the address is devoted to the careful description of a central council as a controlling authority for the voluntary hospitals; and if any additional aid is necessary it is submitted that the better system would be a grant-in-aid system worked from such a central council, as a means of obviating the interference of local authorities or the putting the institutions on the rates.

The idea of a central controlling board for hospitals is not new; such a council was recommended by the House of Lords in 1892. "The three great funds already exercise, if not *de jure*, certainly *de facto*, definite and distinct powers in relation to the voluntary hospitals to whom their grants are made." But the King's Fund has no legal sanction to enforce its dictates, though the power of the purse is great. "The new central council should, if and when instituted, be at once invested with full and general advisory and supervisory powers, and not mainly, as in the case of the King's Fund, with powers for the collection and distribution of money." It is the extra money so badly needed (over and above the grants) which is so hard to get, while the apprehension of further diminution in the contributions of the charitable is continually present to damp the enthusiasm of those responsible for management and extension.

#### The Aims of the Council.

The scheme of control by a central council outlined by Mr. Buchanan includes among other provisions the following:—

All patients should be encouraged or made to pay all they can afford towards the cost of the benefits they receive; and this money should be collected and paid over by the "Public Registrar" of the proposed Public Assistance Authority into the funds of the hospital which has done the work for the patients.

The provident dispensary should be a kind of court of first instance, introduced and maintained by the Public Assistance Authority. It should reduce the number of out-patients and increase the work of and benefit the general practitioners.

All the *working* members of hospital committees should be at first retained and from the central council there should be co-opted others who, with the original body, will at first form the committee of the hospital; the full committee of each hospital is not to exceed seven in number, of whom two should be medical men of the consulting staff.

#### Direct Representation of Hospitals on the Council.

One or two representatives to be elected from the hospital committee on to the central council, which would perform many of the functions of the general courts of governors, etc. It will usually be found that there are not more than five working members on hospital committees.

The new council should include the King's Fund council and the other great funds—the Hospital Sunday Fund and the Hospital Saturday Fund.

Voluntary hospitals should remain independent of the Public Assistance or Poor Law Authority as a supplementary service.

* *The Functions of the Voluntary Hospitals in Relation to the Proposed Public Assistance Authority.* By J. C. Buchanan. London: Bradbury and Agnew. Price 1s. net.

Lay secretaries should be retained for administration. Their status and prospects are likely to improve and to be such as would attract first-class men to the service.

The work of classifying should be done by the Public Assistance Authority in the provident dispensaries, and only patients should attend at the hospital who are recommended by either the dispensary doctor or their own medical attendant.

Even when the voluntary hospital has been relieved of the majority of the cases which should never attend at all, it is likely that there will be a deficit at the end of the year due to decrease in the amount of subscriptions, and here the central council will step in to take the matter in hand. After enquiry they should have the power to make adequate grants and to initiate reforms.

### Expert Advice.

The central body should have the benefit of the advice of representatives of all professions in dealing with technical matters such as building construction and fittings. There will be quite small subcommittees and an executive committee. The council will also determine what degree of efficiency is to be considered satisfactory.

All buying is to be done by one department conducted by experts and all advertising to be arranged by the finance committee to avoid wasteful competition for funds. Subscriptions are still to be received by the individual hospitals as well as by the central council.

### The Example of the King's Fund.

It will be seen that in a great measure this scheme simply embodies an amplification of the powers of the King's Fund, which has already done much in preparing the way for the advent of a central controlling force. It has powers of inspection and tests proposed reforms by committees of enquiry. It has brought into almost general use a simplified system of uniform accounts and in other ways advanced the interests of the voluntary hospitals. At present the hospitals are practically exempt from outside control except for the three great funds, and it appears a natural corollary that the controlling force should be evolved out of a combination of these funds, already intimately connected. Relations between the voluntary hospitals are more friendly and co-operation is nearer the range of possibility than was the case twenty years ago.

### Co-operation and Municipal Aid.

We do not think that the suggestion of a central department for the purchasing of supplies will be generally acceptable or found to work so advantageously as its advocates expect, but apart from this subsidiary item, the scheme outlined above is one that should certainly make for efficiency in the hospital world. The individual characteristics of the several hospitals will still have play and the responsibility of the managers will not be too greatly lessened; but a material relief from anxiety as to funds and the tighter control by an expert authority will foster emulation and a more hopeful spirit.

The author of the pamphlet points out also that the institution of a Central Hospital Board would be a standing obstacle to the municipalisation of hospitals if ever financial distress made it necessary for them to have recourse to either Government aid or municipal subvention, and "more particularly, if housed in fine offices, would be an outward and visible sign of the public wish to preserve the voluntary system which is so much cherished."

### Superintendents: Lay or Medical?

The question whether it is better to have a lay secretary at the head of affairs in a large hospital or to place the control in the hands of a medical superintendent is one on which Mr. Buchanan must naturally be biased and would decide in favour of the lay secretary. The point is mainly one of getting the right sort of man to fill such a difficult position, and while we are inclined to think that the advantages in having a medical man at the head of a hospital are the greater, it must, nevertheless, be admitted that the chances of getting a really suitable man from the medical profession are considerably less than in the alternative case.

### The Limitation of Hospital Work.

One of the most important sections of the address deals with the question of relieving the hospitals from the encumbrance of so many trivial and unsuitable cases. It is hoped that in time the outpatient departments will become almost purely consultation departments, and that patients will only be admitted on the recommendation of a medical man. This question and that of the extension of the provident dispensary system and the relation of the Poor Law infirmary to the teaching hospital on the one hand and to the dispensary on the other, are also considered by Mr. Buchanan in a most interesting manner. For the present we must leave these and other matters so ably discussed in this instructive pamphlet, which is well worth the perusal not only of administrative officials of hospitals but also of medical men in general practice.

